# New Insights into the Ageing of Linseed Oil Paint Binder: A Qualitative and Quantitative Analytical Study

**DOI:** 10.1371/journal.pone.0049333

**Published:** 2012-11-14

**Authors:** Ilaria Bonaduce, Leslie Carlyle, Maria Perla Colombini, Celia Duce, Carlo Ferrari, Erika Ribechini, Paola Selleri, Maria Rosaria Tiné

**Affiliations:** 1 Dipartimento di Chimica e Chimica Ind., Università di Pisa, Pisa, Italy; 2 Departamento de Conservação e Restauro, FCT/UNL, Caparica, Portugal; 3 National Institute of Optics (INO) del CNR, Pisa, Italy; Northeastern University, United States of America

## Abstract

This paper presents an analytical investigation of paint reconstructions prepared with linseed oil that have undergone typical 19th century treatments in preparation for painting. The oil was mechanically extracted from the same seed lot, which was then processed by various methods: water washing, heat treatments, and the addition of driers, with and without heat. A modern process lead white (Dutch source, Schoonhoven) and a commercially available vine black were used as pigments. The reconstructions were prepared in 1999, and naturally aged from then onwards. We compared thermogravimetric analysis (TG), which yields macromolecular information, with gas chromatography-mass spectrometry (GC-MS) and direct exposure mass spectrometry (DEMS), which both provide molecular information. The study enabled us to quantitatively demonstrate, for the first time, that the parameters used to identify drying oils are deeply influenced by the history of the paint. In particular, here we show that the ratio between the relative amounts of palmitic and stearic acid (P/S), which is used as an index for differentiating between drying oils, is extremely dependent on the pigments present and the age of the paint. Moreover the study revealed that neither the P/S parameter nor the ratios between the relative amounts of the various dicarboxylic acids (azelaic over suberic and azelaic over sebacic) can be used to trace the sorts of pre-treatment undergone by the oil investigated in this study. The final results represent an important milestone for the scientific community working in the field, highlighting that further research is still necessary to solve the identification of drying oils in works of art.

## Introduction

Traditional linseed oil based paint consists of a heterogeneous mixture of organic and inorganic compounds, frequently arranged in a complex multi-layered structure. Initial chemical reactions involved in the drying of fresh oil paint are mainly the result of autoxidation [1], 2,3,4 in which a dry film results from cross-linking reactions taking place between the triacylglycerols. During drying and ageing, further chemical changes occur: hydrolysis of the ester bonds, formation of new oxygen containing functional groups, oxidative cleavage of the fatty acid hydrocarbon chains, and metal-ion coordination of the fatty acid groups of the cross-linked material and non cross-linked fractions [1], [5], [6]. Several phenomena can take place during drying and ageing as a result of the interaction of the binder with the pigments. Cations in certain pigments (such as zinc, copper and lead containing pigments) commonly react with free fatty acids present in the paint [7]: the conversion of the fatty acid groups into the corresponding metal carboxylate anion is, in fact, a thermodynamically favoured process [8] and leads to the formation of insoluble metal soaps, which have been extensively documented and investigated [9], [10], [11].

The nature of the pigment present appears to have a crucial role in the chemical composition of an aged oil paint [12], and is often responsible for some well known degradation phenomena [13], [14], [15], [16], [17], [18], [19], [20]. It is also believed that the pre-treatment undergone by the oil [21], [22] to prepare it for paint making has an influence in determining its ageing pathway, and can be traced by chemical analysis of the paint binder [23], [24]. For example it is reported in the literature that double bonds isomerise when the oil is thermally treated [25], [26], and, as a consequence, the relative amounts of suberic and sebacic acids increase with respect to azelaic acid [6], [23], [24], [27].

How oil processing can affect the way a paint behaves during its application has been investigated [21], [28], but to what extent such oil processing can influence the composition of an aged paint is still not fully clear. The first aim of this study therefore is to understand if the type of pre-treatment undergone by the oil influences the ageing pathway of the paint film. In addition we examine whether it is possible to recognise the type of pre-treatment from a chemical analysis of a paint sample. We investigated paints that had been prepared with linseed oil after the oil had undergone various pre-treatments in use throughout the nineteenth century [22]. The oil was mechanically extracted from the same lot of linseeds then processed by different methods: water washing, heat treatments, and the addition of driers, with and without heat [21]. The paints had been prepared using four different pigments: for this investigation a lead white (D), and a vine black (B) were chosen, the former for its well known reactivity in oil, and the latter as its held to be relatively inert in an oil binder. GC-MS, DEMS, and TG were used for the investigation. This work is the follow-up to a previous study characterizing the oil alone which revealed how pre-treatments produce binders with different physical-chemical qualities [28].

**Table 1:** Description of the paints analysed.

name of thepaint sample	treatment undergone by the freshlypressed linseed oil	pigment		% oil by weight*
		lead white - ZD series	vine black - ZB series	
ZD	*no treatment (Z)*	+		13.8
ZB			+	38.3
XD	*washing in purified water (X)*	+		20.6
XB			+	41.3
ZH150D	*heating up to 150°C (ZH150)*	+		13.8
ZH150B			+	41.5
ZH300D	*heating up to 300°C (ZH300)*	+		19.3
ZH300B			+	43.0
A2D	*shaking with lead (II) oxide (A2) (room* *temperature)*	+		17.2
A2B			+	39.0
AH2D	*heating up to 150°C with lead (II) oxide (AH2)*	+		13.8
AH2B			+	39.0
B8D	*shaking with lead acetate (B8) (room* *temperature)*	+		17.2
B8B			+	40.3
B8HD	*heating up to 150°C with lead acetate (B8H)*	+		20.2
B8HB			+	41.0
C8D	*shaking with lead subacetate (C8) (room temperature)*	+		21.3
C8B			+	42.3
C8HD	*heating up to 150°C with lead subacetate* *(CH8)*	+		19.5

* % oil content of the paint.

TG has been successfully applied in the field of cultural heritage to study a wide range of different materials [28], [29], [30], [31], [32], [33], [34], and, in this paper, we use it to systematically investigate and compare the thermal behaviour of oil paints made with a single source of oil pre-treated in different ways. TG enabled us to assess the effects of the two pigments on the ageing of the oil binder. Moreover we present for what we believe is the first time, the TG characterisation of metal soaps in lead white containing paints. By means of quantitative evaluation of the relative amounts of binder/pigments, important conclusions are drawn on the parameters used to identify and characterise drying oils, such as the ratio between the relative amounts of palmitic and stearic acids (P/S) [1], and the various di-carboxylic acids, that is azelaic over suberic (A/Sub) and azelaic over sebacic (A/Seb) [6], [24], [27]. Finally the effects of oil pre-treatments on the paint’s chemical composition are discussed.

**Figure 1:**
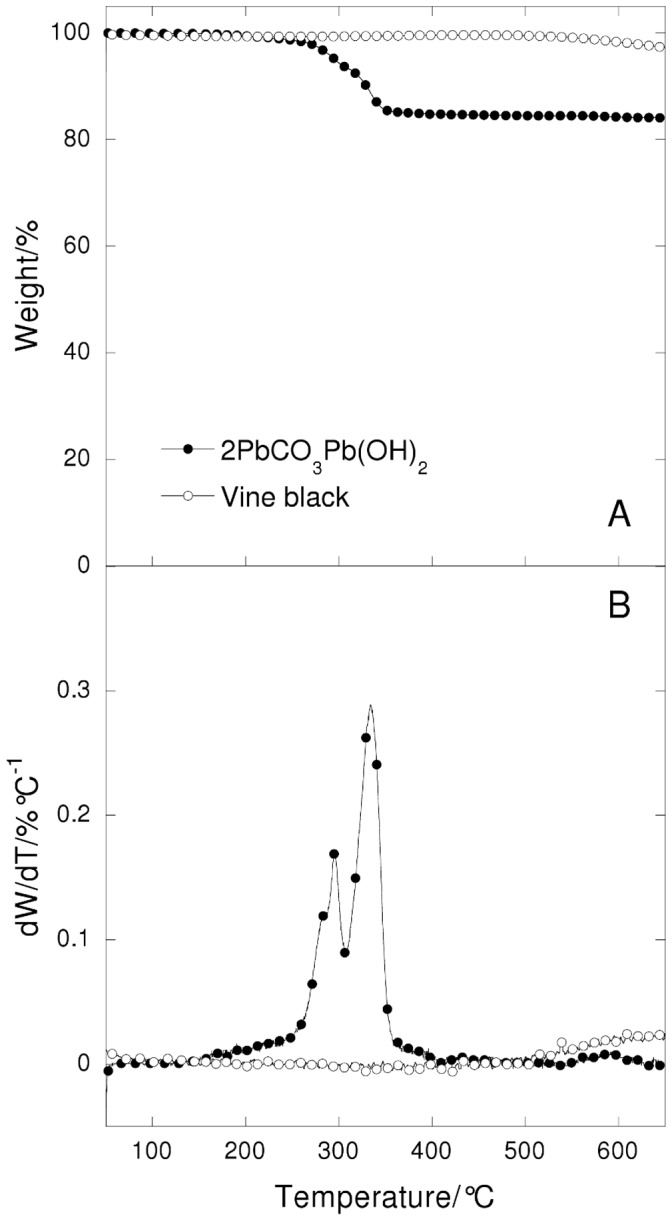
Thermogravimetric curves (A) and their derivatives (B) under nitrogen flow of lead white and vine black pigments at 20°C/min heating rate in function of temperature.

## Materials and Methods

### Reagents

All solvents were Baker HPLC grade and were used without any further purification. *N*,*O-*bis(trimethylsilyl)trifluoroacetamide (BSTFA) was purchased from Sigma-Aldrich (USA).

The following solutions were prepared by weighing pure substances and were used as standards: tridecanoic acid solution in isooctane (Sigma-Aldrich (USA), purity 99%), 135.48 *µ*g/g, was used as a derivatization internal standard; hexadecane solution in isooctane (Sigma-Aldrich (USA), purity 99%), 80.34 *µ*g/g, was used as an injection internal standard; mono and dicarboxylic acids solution in acetone, containing lauric acid (0.24 mg/g Lau), suberic acid (0.27 mg/g, Sub), azelaic acid (0.28 mg/g, A), myristic acid (0.25 mg/g, Myr), sebacic acid (0.3 mg/g, Seb), palmitic acid (0.25 mg/g, P), oleic acid (0.51 mg/g, O), and stearic acid (0.51 mg/g, S) were used for the quantitation of the different compounds in the chromatograms. All acids, purity 99%, were purchased from Sigma-Aldrich (USA).

**Figure 2:**
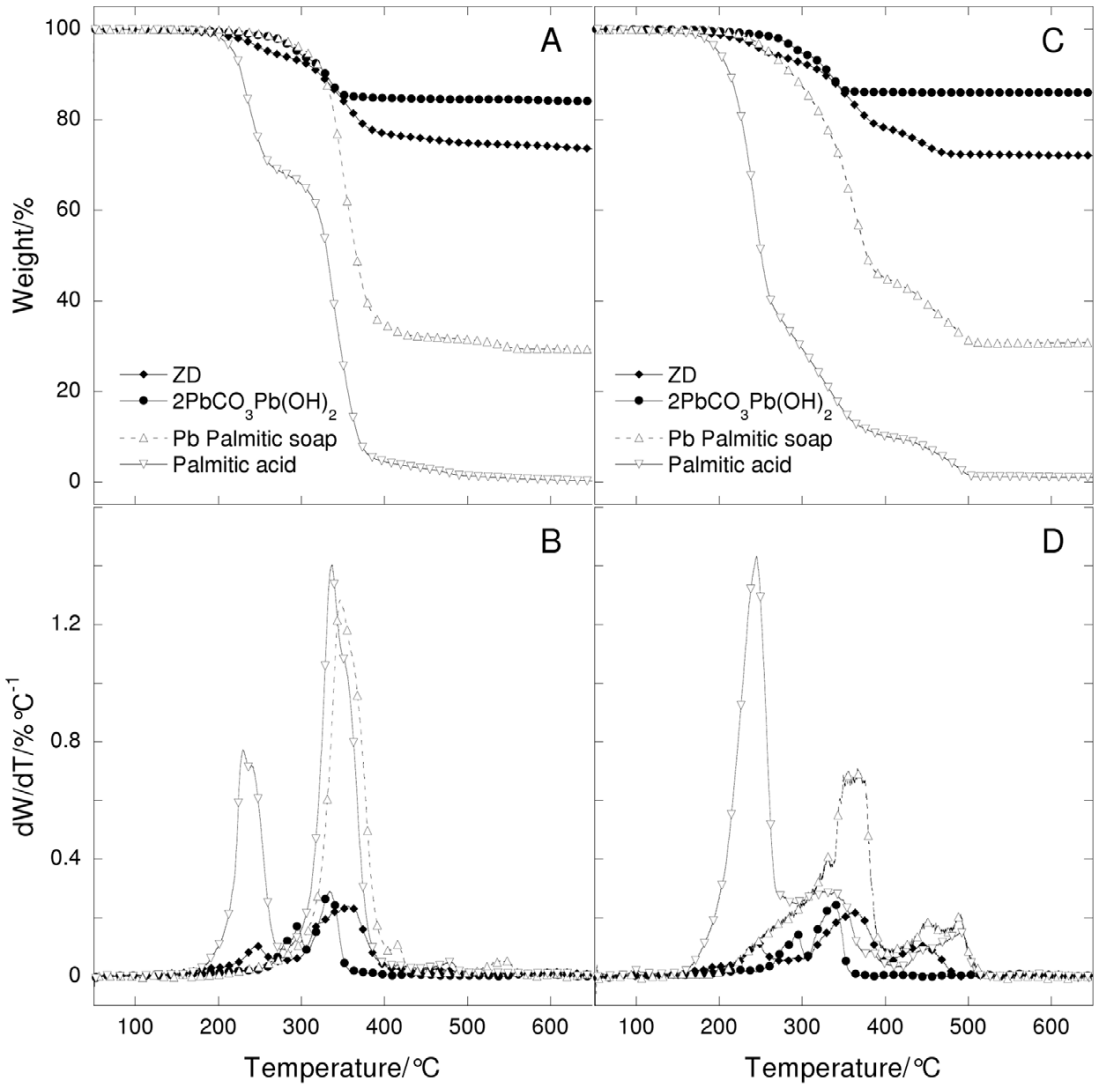
Thermogravimetric curves and their derivatives under nitrogen flow (A,B) and under air flow (C, D) at 20°C/min heating rate as a function of temperature of ZD paint (ZD = Z untreated oil + lead white) and Pb palmitate, lead white and palmitic acid.

### Standards

Stearic acid, DL-α-Palmitin, 1,2-Dipalmitolyl-glycerol, Tripalmitin, were supplied by Sigma-Aldrich (USA), all with a purity >99%. Lead stearate and lead palmitate were prepared following the synthesis reported in the literature [8].

**Figure 3:**
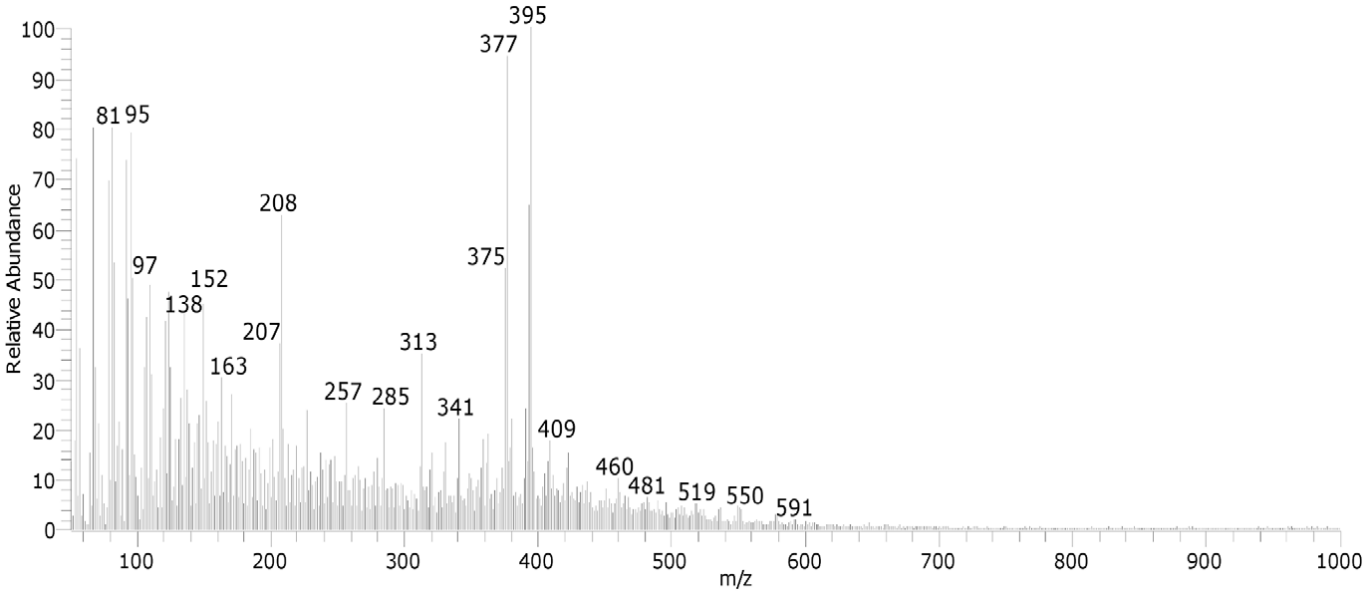
The DE-Mass spectrum of the ZD (Z oil + lead white) in CH_4_ chemical ionisation mode.

### Apparatus

- A microwave oven model MLS-1200 MEGA Milestone (FKV, Sorisole, Bergamo, Italy) was used for the saponification of the glycerolipids. Operating conditions were as follows: 80°C, 200 W, 60 min.
- 6890N GC System Gas Chromatograph (Agilent Technologies), coupled with a 5973 Mass Selective Detector (Agilent Technologies) single golden quadrupole mass spectrometer, equipped with a PTV injector. The mass spectrometer was operated in the EI positive mode (70 eV). The MS transfer line temperature was 280°C; the MS ion source temperature was kept at 230°C and the MS quadrupole temperature at 150°C. For the gas chromatographic separation, an HP-5MS fused silica capillary column (5%diphenyl-95%dimethyl-polysiloxane, 30 m×0.25 mm i.d., 0.25 µm film thickness, J&W Scientific, Agilent Technologies, Palo Alto, CA, USA) coupled with a deactivated silica pre-column (2 m×0.32 mm i.d., J&W Scientific Agilent Technologies, Palo Alto, CA, USA) using a quartz press fit was used. The carrier gas was used in the constant flow mode (He, purity 99.995%) at 1.2 ml/min. The PTV injector was used in splitless mode at 300°C and the chromatographic oven was programmed as follows: 80°C isothermal for 2 minutes, 10°C/min up to 200°C, 200°C isothermal for 3 minutes, 10°C/min up to 280°C, 280°C isothermal for 3 minutes, 20°C/min up to 300°C isothermal for 30 minutes;

Chromatograms were acquired in both total ion current (TIC) mode and selected ion monitoring (SIM) mode. Quantitative analyses were performed in SIM mode using calibration curves and daily injections of standards to evaluate changes in the response of the instrument.
Analytical procedure [35]: the sample undergoes microwave-assisted saponification/salification with 300 *µ*L of KOH_ETOH_ 10% wt at 60°C for 120 min. After saponification, the alcoholic solution is diluted in bidistilled water and acidified with hydrochloric acid (aqueous solution 1∶1). The acidic solution is then extracted with hexane (200 *µ*L, three times) and, afterwards, with dieyhyl ether (200 *µ*L, three times). An aliquot of the organic solution is admixed with 5 *µ*L of tridecanoic acid solution (derivatisation internal standard), evaporated to dryness under a nitrogen flow and derivatised with 20 *µ*L of N,O-bis(trimetilsilil)trifluoroacetammide (BSTFA), 200 *µ*l of isooctane (solvent) at 60°C for 30 min. Lastly 5 *µ*L of hexadecane solution are added. A total of 2 *µ*L of the isooctane solution is analyzed by GC/MS.

- *DE-(CI)MS*: The sample (a few nanograms) was placed on the rhenium filament at the end of a probe. The heating of the probe was operated in current programmed mode, with a maximum current of 1000 mA, which corresponds to approximately 1000°C. The conditions to obtain a total ion current (TIC) curve as a function of time were obtained by programming the probe as follows: 0 mA for 20 s, from 0 mA to 1000 mA in 2 s, then 1000 mA for 60 s. A mass spectral fingerprint was obtained by averaging the mass spectra in the desired time range. The desorbed material was ionized by chemical ionisation (methane, 1.0 mL s^−1^) in a Thermo Finnigan Polaris Q ion trap mass spectrometer (ion source temperature of 250°C), and a total ion current was acquired as a function of time. The mass spectrometer was scanned over an m/z range of 50–1000.
- A Perkin Elmer Thermobalance model TGA7 was used. Two kinds of experiment were carried out: a) isothermal experiments at 80°C under a constant flow (90 ml/min) of air; b) dynamic experiments at a constant rate of 20°C/min, from 50°C to 700°C. Measurements were performed under a constant flow (90 ml/min) of air or nitrogen. All the curves were normalised by subtracting the background (the empty crucible). The amount of sample in each measurement varied between 1.7 and 2.4 mg.

**Figure 4:**
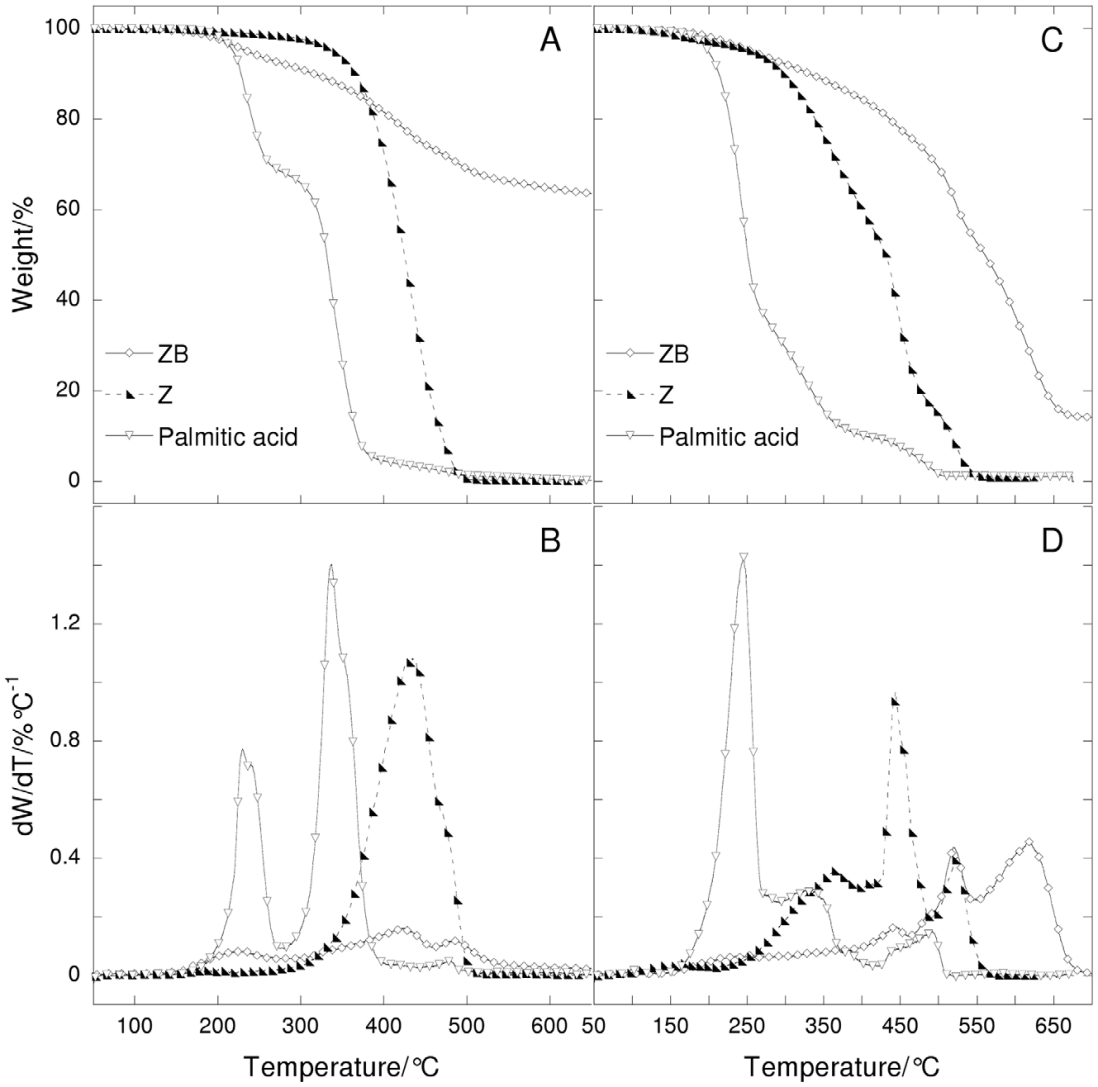
Thermogravimetric curves and their derivatives under nitrogen (A,B) and air flows (C, D) at 20°C/min heating rate as a function of temperature of a ZB paint (Z untreated oil + vine black) and some related reference materials (Z oil and palmitic acid).

### Samples

The study was performed on hand-ground paint samples prepared in 1999 as part of the MOLART Programme [21] using historical recipes for oil pre-treatments published in Britain in the 19^th^ century [22]. The oil was extracted mechanically using a custom built stainless steel oil press supplied by Hayo de Boer. Linseeds were from the same seed lot organically grown in the Netherlands [21], [36].

**Figure 5:**
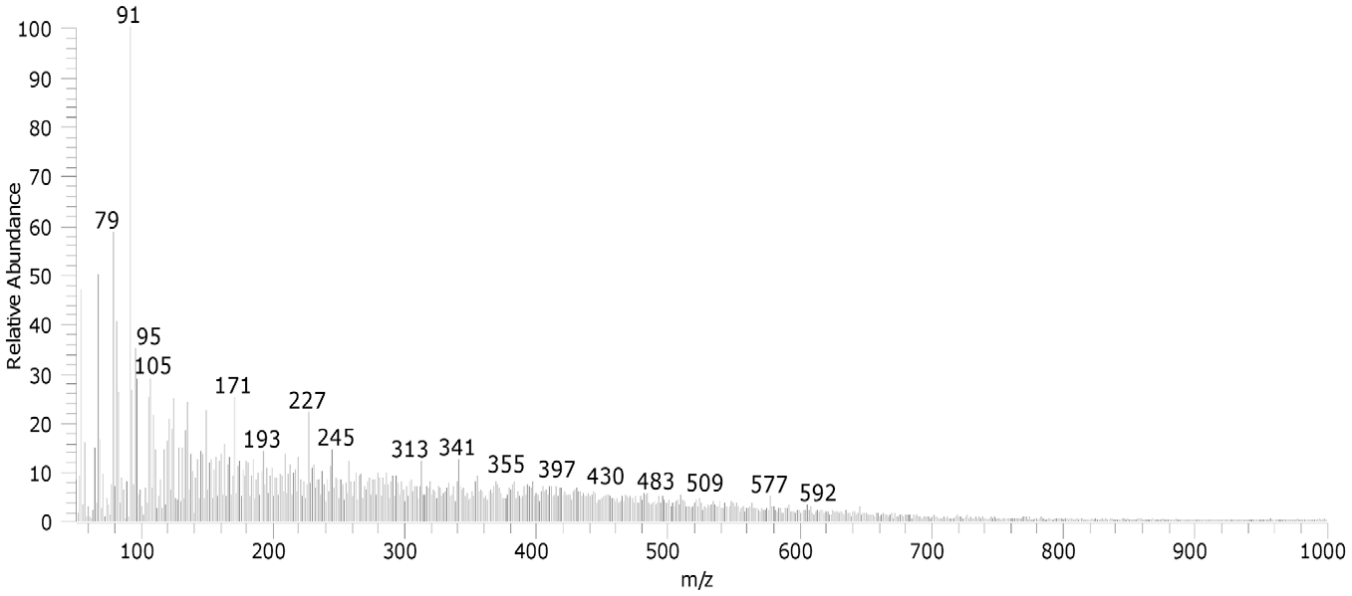
The DE-Mass spectrum of the ZB paint (Z oil + vine black) obtained in CH_4_ chemical ionisation mode.

Two modern sources of lead white, a vine black and an umber were used to make paints with the differently processed oil (detailed in the Molart Report reference [21]. For this study the paints made with the Dutch source (Schoonhoven) lead white (D), and the vine black (B) from Kremer Pigmente were investigated. The paints had been applied to various substrates including polyester film (Melinex®) where a fixed-thickness applicator was used to achieve a uniform paint film (called a “draw-down”). For this investigation paint samples were taken from the draw downs which had been stored at ambient temperatures in a variety of light conditions (dark storage and light exposed) for approximately 11 years. Table 1 lists the paint analysed.

In addition to the samples of the ZD and ZB series, the liquid Z oil alone (untreated oil, extracted 1999, kept in a closed bottle in the darkness), and a film of Z oil alone which had been applied to a glass slide in 2003 was analysed and is referred to here as, “unpigmented dried oil”.

**Table 2:** Characteristic parameters determined for the paint samples.

oil pre-treatment codes	P/S			A/P			∑D			A/Sub			A/Seb		
	no pigment	lead white	vine black	no pigment	lead white	vine black	nopigment	lead white	vine black	nopigment	lead white	vine black	no pigment	lead white	vine black
**Z**	1.5	1.3	1.3	1.5	2.8	2.1	40.7	66.9	53.4	15.3	5.0	7.4	8.9	10.7	28.8
**ZH150**		1.3	1.3		1.4	1.7		50.7	56.8		5.3	5.8		9.3	30.7
**ZH300**		1.4	1.5		1.3	1.1		41.6	46.6		6.3	5.7		9.1	17.8
**A2**		1.3	1.3		2.3	1.3		60.1	50.8		5.5	7.1		11.7	24.5
**AH2**		1.5	1.2		3.0	1.9		67.1	58.0		7.9	6.1		16.1	14.4
**B8**		1.5	1.7		1.1	1.4		43.7	49.5		10.2	6.5		9.0	24.1
**BH8**		1.5	1.6		1.2	1.0		45.6	42.0		5.5	6.2		15.0	28.9
**C8**		1.3	1.4		2.1	2.8		51.5	44.6		5.6	5.5		9.5	22.0
**CH8**		1.3			2.1			43.9			6.6			17.3	

## Results and Discussion

### Characterisation of Pigments

The lead white and vine black thermogravimetric curves under nitrogen flow are reported in Figure 1.

The decomposition of the lead white pigment powder shows two main steps that correspond to the loss of water at 280°C, followed by the loss of carbon dioxide at 340°C [3]. After 400°C there is a plateau. By the end of the degradation the lead white powder had converted completely into lead (II) oxide. The analysis under nitrogen and air flow gave the same results. The total weight loss of the pigment was about 14.4%, in accordance with the literature [33].

**Figure 6:**
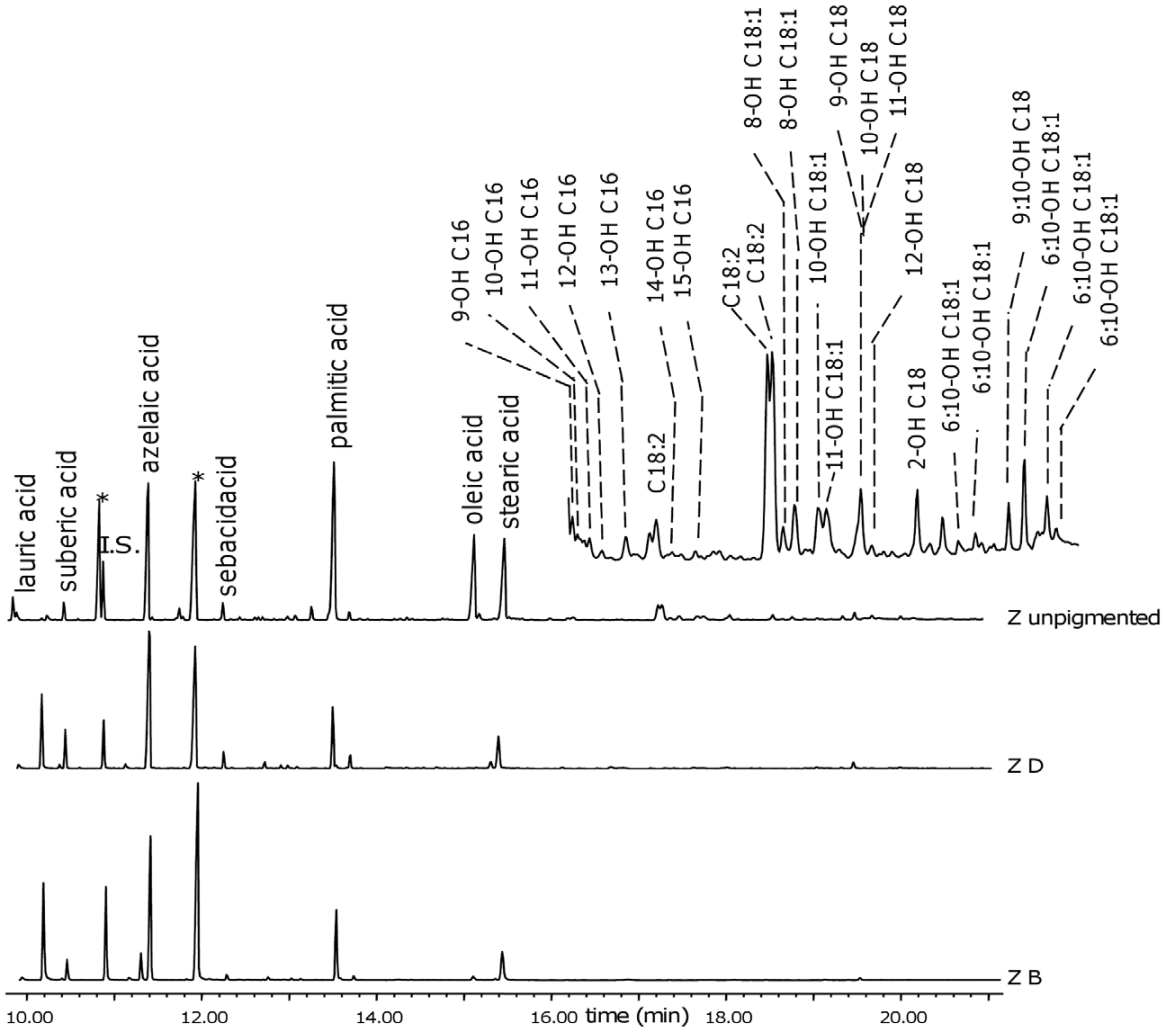
Chromatogram of dried oil film (without pigment) compared to samples ZD and ZB. I.S.: internal standard; n-OH C Nr: n-hydroxy linear saturated monocarboxylic acid with Nr carbon atoms. n-OH C Nr:M: n-hydroxy linear monocarboxylic acid with Nr carbon atoms and M unsaturations.

The vine black pigment powder shows a broad weight loss above 500°C that can be ascribed to the decomposition of lignin, which makes up 4–35% of most wood biomass. The total weight loss observed for vine black pigment was found to be 4%. Accordingly, most (96%) of the vine black pigment is pyrolised char matter [37].

### Characterisation of the Paint Samples

#### *Oxidation and hydrolysis of linseed oil in the paint reconstructions*

The nature of the pigment strongly influenced the thermal stability of the paint.

The thermogravimetric curves of sample ZD (lead white paints) are shown in Figure 2, in comparison with lead white powder (86% of the initial weight of the paint layer ZD, as shown in Table 1), lead palmitate and palmitic acid.

**Table 3:** Experimental and calculated^2^ final weight of the paint samples of the ZD series at 700°C.

paint sample codes	lead white addedto the oil (%)	expected weight of thepaint at 700°C (%)	experimental weight of thepaint at 700°C (%) (N_2_)	experimental weight of the paint at 700°C (%) (air)
XD	81	69	74	69
ZD	86	74	74	74
ZH150D	79	68	68	70
ZH300D	86	74	76	75
A2D	83	71	67	65
AH2D	86	74	74	74
B8D	88	71	74	72
BH8D	80	68	73	69
C8D	79	67	70	67
CH8D	80	69	73	71

The thermogram of the lead white paint shows many steps: the first step at 245°C, the second step at a higher temperature (350°C) and other steps are observed above 400°C. The total weight loss of the lead white paint is about 26.6%. The position of the steps (temperatures of weight losses) and their shape in the thermograms clearly indicate that the ZD paint contains free fatty acids (step at 230°C) and carboxylates (step at 350°C), while the lead white pigment is not visible. This means that the binder is hydrolysed, although it is not clear whether the hydrolysis is complete, or whether some glycerides are still present. Under nitrogen flow the thermograms of tripalmitine, dipalmitine and tripalmitine show a main weight loss centred at about 420°C [28]: these steps cannot be seen in the thermogravimetric curve of ZD paint because at the same temperature a weight loss of lead palmitate takes place.

**Table 4:** Experimental and calculated3 weight loss of the paint samples in the ZB series at 700°C.

paint reconstruction	expected oil content of thepaint reconstruction (%)	expected weight loss of thepainting replica at 700°C (N_2_) (%)	experimental weight loss of thepainting replica at 700°C (N_2_) (%)
ZB	38	40	36
XB	41	43	35
ZH150B	42	44	38
ZH300B	43	45	40
A2B	39	41	35
AH2B	39	41	35
B8B	40	42	35
BH8B	41	43	36
C8B	42	44	36

These observations are confirmed by DE-MS data. The DE-Mass spectrum of sample ZD is reported in Figure 3. The main peaks at m/z 395 and m/z 377 correspond to the lead azelate minus one fatty acid radical (the [Pb(C_8_H_15_COO)]^+^), and followed by the loss of a water molecule, respectively. In the spectrum, the molecular ions at m/z 257 and 285 can be ascribed to protonated free fatty acids. The fragment ions at m/z 138 and 152 are attributable to suberic and azelaic acid fragmentation. This all indicates an oxidized paint layer, and also that the saponification of fatty acids has taken place over time. The cluster ions at m/z 570–578, which can be ascribed to the fragment ions formed from triglycerides due to the loss of an acylium group ([M-RCOO]^+^) [38], are not present, indicating that diglycerides and triglyecerides, if present, are not abundant in the paint film. On the other hand, the peaks at m/z 341 and 313, corresponding to the fragment ions ([RCO+74]^+^) with the glycerol backbone minus one hydroxyl group, are still relatively abundant in the mass spectrum, indicating that monoglycerides are still present. These results confirm the hypothesis that the oil binder has undergone hydrolysis as previously discussed above, based on the TG data.

**Figure 7:**
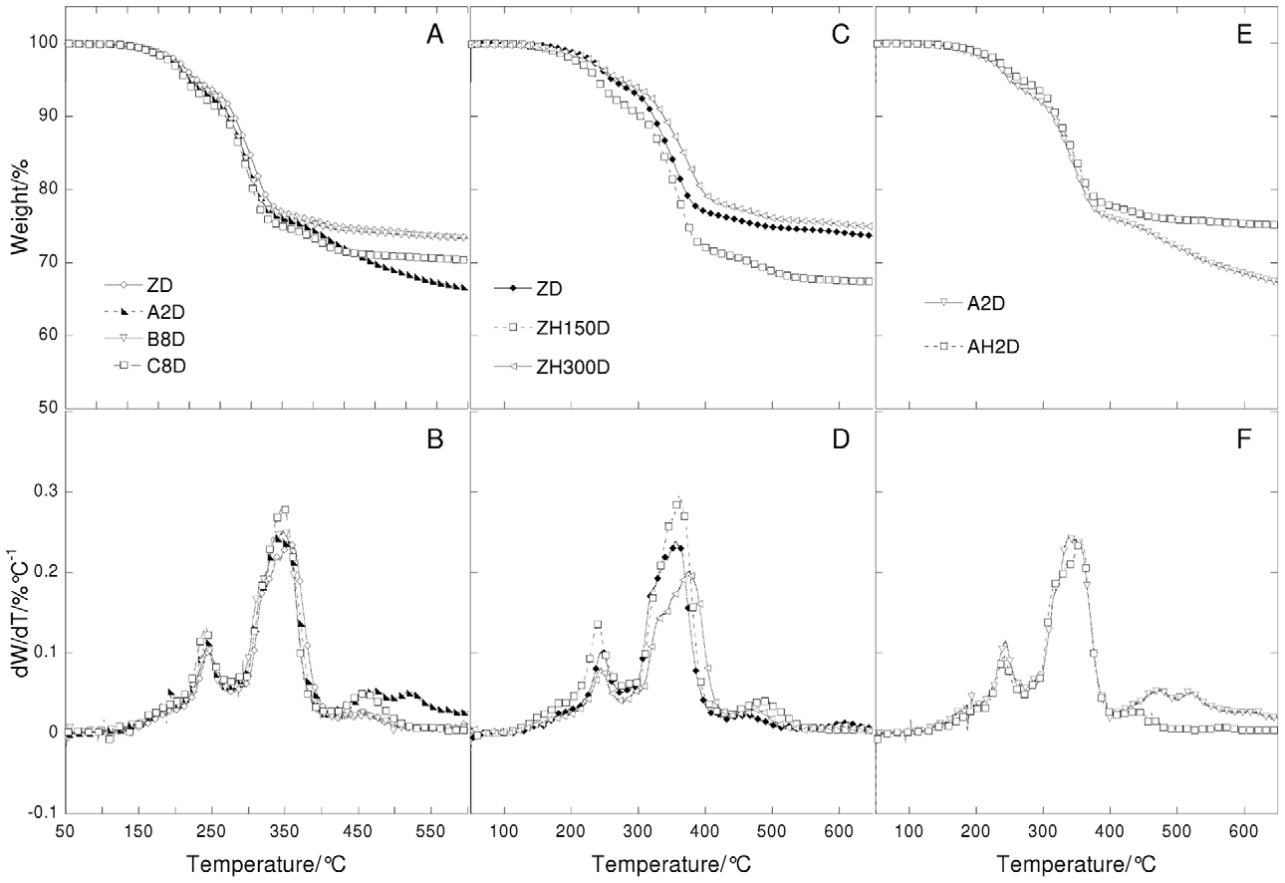
Thermogravimetric curves (A, C and E) and their derivatives (B, D, and F) under nitrogen flow at 20°C/min heating rate in function of temperature of lead white paint layers. Left: paint reconstructions prepared with linseed oil treated with different lead based driers (ZD = untreated; A2D = Z oil treated with litharge; B8D = Z oil treated with lead acetate; C8D = Z oil treated with lead subacetate). Centre: paint reconstructions with heated linseed oil (ZD = Not heated oil; ZH150D = Z oil heated to 150°C; ZH300D = Z oil heated to 300°C). Right: paint reconstructions prepared with linseed oil treated with litharge with and without heat (A2D = Z oil treated with litharge not heated; AH2D = treated with litharge and heated at 150°C).

**Figure 8:**
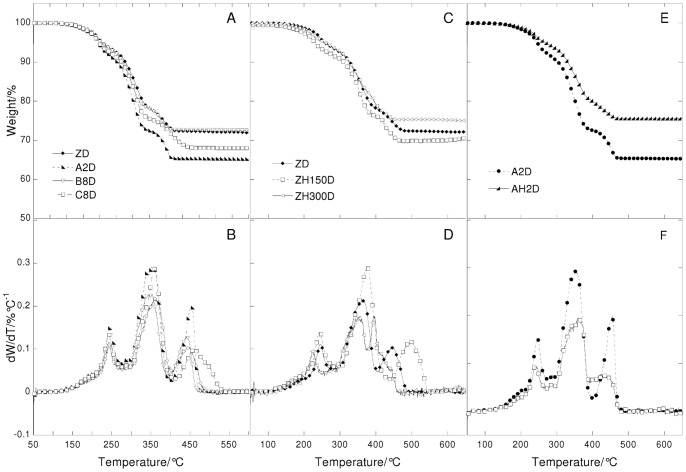
Thermogravimetric curves (A, C and E) and their derivatives (B, D, and F) under air flow at 20°C/min heating rate in function of temperature of lead white paint layers. Left: paint reconstructions prepared with linseed oil treated with different lead based driers (ZD = untreated; A2D = Z oil treated with litharge; B8D = Z oil treated with lead acetate; C8D = Z oil treated with lead subacetate). Centre: : paint reconstructions with heated linseed oil (ZD = Not heated oil; ZH150D = Z oil heated to 150°C; ZH300D = Z oil heated to 300°C). Right: paint reconstructions prepared with linseed oil treated with litharge with and without heat (A2D = Z oil treated with litharge not heated; AH2D = treated with litharge and heated at 150°C).

**Figure 9:**
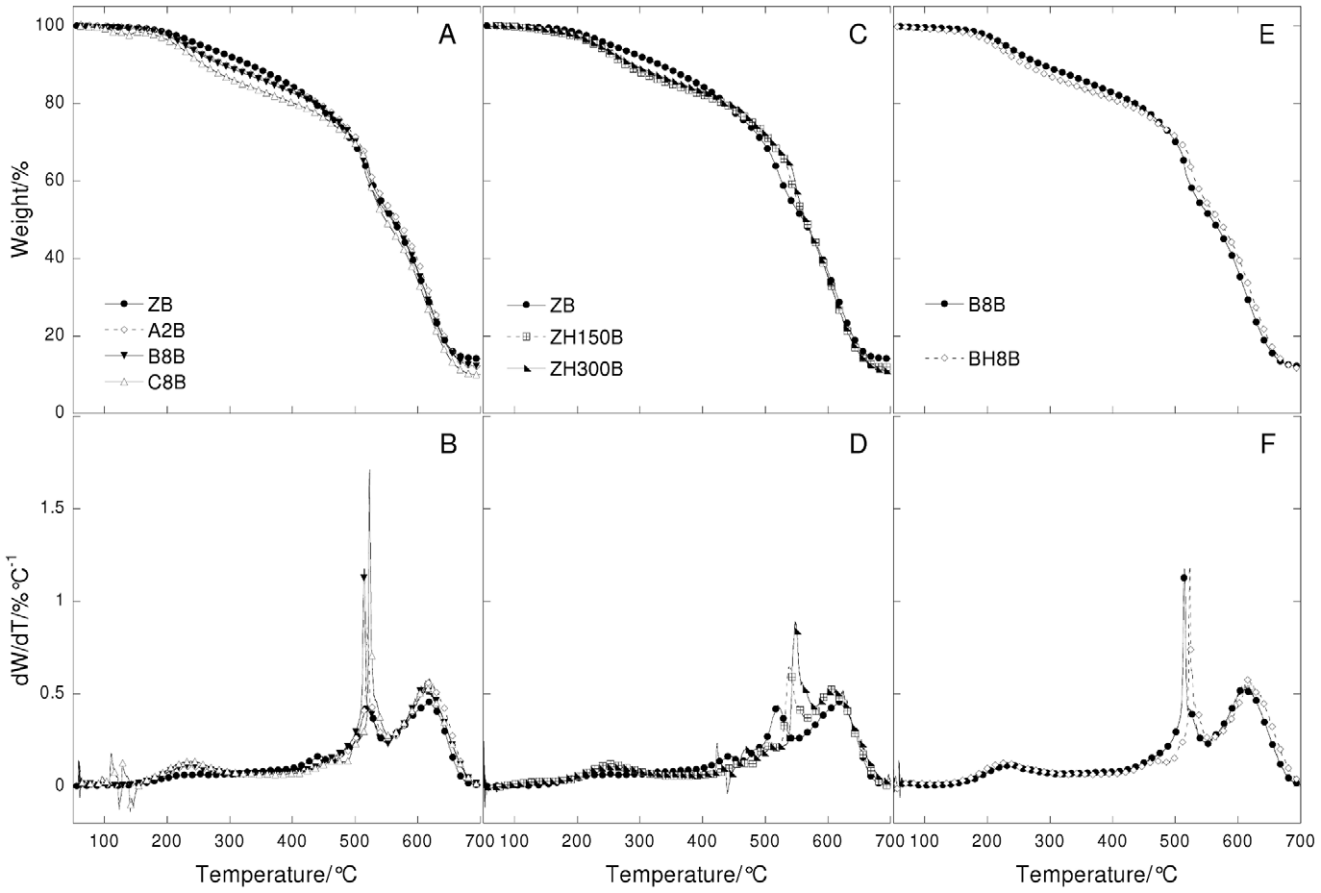
Thermogravimetric curves (A, C and E) and their derivatives (B, D, and F) under air flow at 20°C/min heating rate in function of temperature of vine black paint layers. Left: paint reconstructions prepared with linseed oil treated with different lead-based driers (ZB = untreated; A2B = Z oil treated with litharge; B8B = Z oil treated with lead acetate; C8B = Z oil treated with lead subacetate). Centre: paint reconstructions with heated linseed oil (ZB = Not heated oil; ZH150B = Z oil heated to 150°C; ZH300B = Z oil heated to 300°C). Right: paint reconstructions prepared with linseed oil treated with lead acetate with and without heat (B8B = Z oil treated with lead acetate; BH8B = Z oil treated with lead acetate and heated at 150°C).

**Figure 10:**
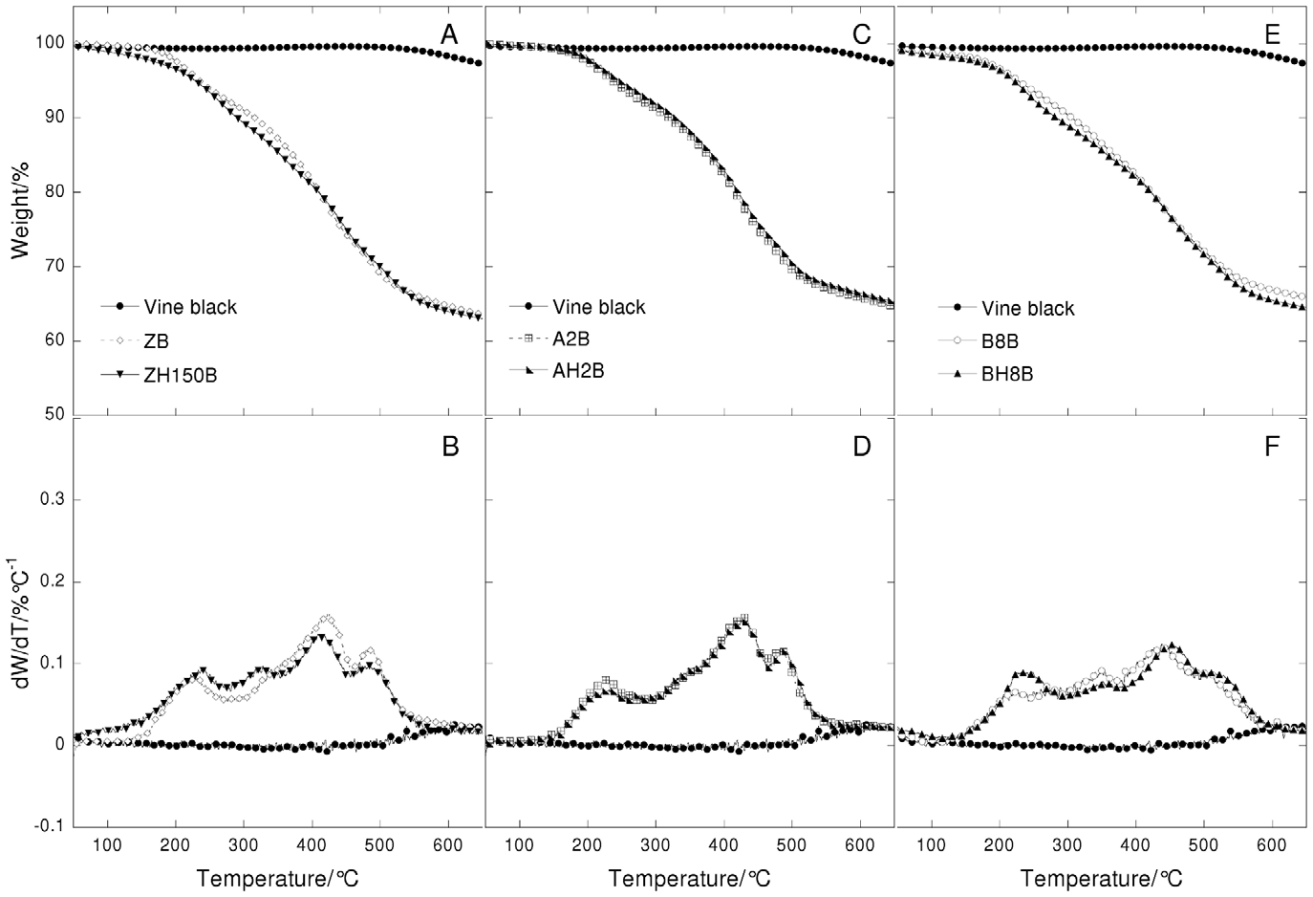
Thermogravimetric curves (A, C and E) and their derivatives (B, D, and F) under nitrogen flow at 20°C/min heating rate in function of temperature of vine black pigment and vine black paint layers. Left: Vine black pigment and paint reconstructions prepared with heated linseed oil (ZB = Not heated oil; ZH150B = Z oil heated to 150°C). Centre: Vine black pigment and paint reconstructions prepared with linseed oil treated with litharge with and without heat (A2B  = Z oil treated with litharge; AH2B = Z oil treated with litharge and heated at 150°C). Right: Vine black pigment and paint reconstructions prepared with linseed oil treated with lead acetate with and without heat (B8B = Z oil treated with lead acetate; BH8B = Z oil treated with lead acetate and heated at 150°C).

The thermogravimetric curves obtained from sample ZB (vine black paints) are reported in Figure 4, in comparison to the Z liquid oil and palmitic acid.

The thermogram of the vine black paint recorded under nitrogen shows three steps: the first step at 225°C, the second step at a higher temperature (420°C) and one more step at 485°C. The total weight loss of the vine black paint is about 36.4%. Thermogravimetric analysis indicates that the ZB paint is clearly partially hydrolysed, confirmed by the two steps observed in the thermograms at high temperature (above 400°C), and at 225°C, which are attributable to free fatty acids, mono and diglycerides [28]. The tail at higher temperatures indicates the formation of high molecular weight compounds caused by cross-linking and oxidation. This is more evident from the results obtained under air flow. The ZB paint thermogram shows at least five steps: steps at 235°C and 335°C, relating to the thermal degradation of free fatty acids, mono and diglycerides [28]; the steps at 440°C and 520°C relating to the thermal degradation of glycerides [28]; and finally, the step at 615°C relating to high molecular weight compounds caused by cross-linking and oxidised compounds.

The degree of hydrolysis of the ZB paint is better understood by using DE-MS analysis (Figure 5). In this case the cluster ions attributable to di- and triglycerides are also absent, while monoglycerides are present. Free fatty acids are not clearly visible (m/z 257 and 285), and the mass spectrum is dominated by fragment ions with low values of m/z. Some of these fragment ions are due to the fragmentation of the aliphatic saturated and unsaturated lipid molecules (m/z 67, 69, 81, 83, 93, 95, 108–109, ….). In addition the base peak at m/z 91 and the peak at m/z 105 are due to alkylated benzenes, which are produced after the pyrolysis of the crosslinked oil paint material [38].

The degree of oxidation of the paints can partly be observed by the results of the GC-MS analyses, shown in **Table 2**, where the characteristic parameters used for the identification of a drying oil are reported.

The oxidation of the paint film is expressed by the A/P (ratio between the relative amount of azelaic and palmitic acid) and the ∑D (sum of the relative amounts of dicarboxylic acids) parameters. The A/P values obtained for the paint samples range between 1 and 3, and ∑D between 40 and 68, versus 0 and 0–6 of liquid linseed oils [28], respectively. In most cases (67% of the samples) the paints with lead white show the highest oxidation level, as testified by the higher A/P and ∑D values. The paints are generally more oxidised than the unpigmented dried oil film, as it can be evidenced in Figure 6 where the total ion chromatogram of the sample of the unpigmented dried oil is compared to those of samples ZD and ZB. The chromatogram of the dried oil film without pigment clearly shows the presence of intermediate oxidation products, such as mono and di-hydroxylated fatty acids, which are not observed in the chromatogram of both of the paint samples.

The fact that most of the paints prepared with lead white are the most oxidised is not surprising, as lead is known to catalyse oxidation reactions [39]. The paints containing vine black are much more oxidised than the dried oil sample alone (without pigment). Aside from the difference in drying time of 4 years, this finding is most likely due to the fact that the dispersion of the pigment particles in the binder increases the contact of the binder itself with oxygen, thus favouring its oxidation.

#### *Double bond transposition*

The double bond transposition taking place during ageing is clearly observed in the chromatogram of the unpigmented dried oil film in Figure 6. Several peaks ascribable to fatty acids with hydroxyl moieties in a number of positions that do not correspond to the original positions of the double bonds can in fact be observed. It is reported that double bonds isomerise when the oil is thermally treated [25], [26], and it is consequently believed that the relative amounts of suberic and sebacic acids increase with respect to azelaic acid [6], [24], [27]. The oils ZH150, ZH300, AH2, BH8, CH8 were all heated. In a previous work [28], in the thermally treated liquid oils kept since the paint samples were prepared in 1999, isomerisation of the double bonds was observed, and the high number of isomers formed indicated that not only cis/trans isomerisation took place, but also double bond transpositions. It is in fact known that unsaturated and mainly polyunsaturated compounds are subjected to oxidation reactions, responsible of the formation of a cross-linked fraction and of degradation products. In particular the uptake of oxygen by double bonds leads to the formation of new oxygen containing functional groups as keto groups, and then to the oxidative cleavage of fatty acid hydrocarbon chains, mainly resulting in the formation of dicarboxylic acids [6], [40]. Despite this, the A/Seb and A/Sub of paints made with thermally treated oils did not show meaningful trends with respect to the non heated oils, even in the case of ZH300, which was the oil heated for longest at the highest temperature. This indicates that double bond transposition is a much more important phenomenon under ageing, than it is as a consequence of the thermal treatment of the oil. This can be explained considering that the ageing of an oil medium is a radical mechanism [3]. These data strongly suggest that A/Sub and A/Seb cannot be used as a general parameter to tell whether the oil used as a binder was pre-heated, alone or in the presence of a lead dryer, as was previously reported [6], [24], [27].

The A/Seb values obtained for the vine black paint are systematically higher than the paints containing lead white. This suggests that double bond transposition taking place during ageing, may be influenced by the presence and nature of the pigment.

#### *Evaporation of the organic medium from the paint layers*

The evaporation of the organic binder from the paints is another extremely interesting aspect that can be observed by comparing the thermogravimetric data of the paint reconstructions with lead white and those with vine black. It is reported in the literature that glycerol is generally not detected in a paint samples [6], [41], because it slowly evaporates from the painting [42], that free fatty acids evaporate easily from a paint film, and that palmitic acid evaporates approximately four times faster than stearic acid [12], [43]. When the paint samples were originally prepared, the relative amounts of pigment and binder were recorded [21]. By comparing the original binder content with the experimental value obtained from the thermogravimetric data, it is possible to estimate the amount of organic matter that evaporated from the paint in the eleven years of natural ageing. In particular the thermogravimetric curve can be used to estimate the relative amount of pigment and paint binder, on the basis of the weight losses observed and knowledge of the chemical transformations undergone by the pigment during the analysis.

#### *ZD series*

A comparison between the expected pigment content and the calculated values of the paints with lead white is reported in Table 3. The first column of Table 3 shows the original content of the pigment (lead white) in the paint. The second colum shows the calculated weight expected after a TGA run to 700°C; the calculation takes account of the weight loss suffered by the pigment alone during a similar TGA scan, as shown in Figure 1, and assumed that all organic matter (the binder) is lost without leaving ash. After heating lead white is in fact transformed into lead oxide. By heating the paint samples containing lead white, we can thus assume that the final weight of the samples is due to the lead oxide formed. The third and fourth columns show the experimental results (final weight) obtained in the presence of nitrogen and oxygen (air) in the TGA scans of the paint; in this case no correction was carried out.

The expected binder content and the values estimated on the basis of the experimental data are quite in agreement with each other, and the discrepancy observed is always within 5%. In most cases the experimental results were slightly higher than predicted, especially under nitrogen. Although this might indicate that a small amount of the oil constituents (free fatty acids and glycerol) evaporated, it is most likely that the higher values obtained under nitrogen could be mostly attributed to the incomplete pyrolysis of the organic fraction taking place at 700°C.

#### *ZB series*

A comparison between the expected pigment content and the calculated values of the paint samples with vine black is reported in **Table 4**. The first column of **Table 4** shows the original content of the binder (oil) added to the pigment (vine black) in the paint. The second column shows the calculated weight loss expected after a TGA run to 700°C; the calculation takes account of the weight loss suffered by the pigment alone during a similar TGA scan, as shown in Figure 1, and assumed that all the oil (the binder) is lost without leaving ash. Vine black is in fact subject to a small weight loss, which is due to the pyrolysis of the organic matter. Thus the final weight observed at the end of the analysis is due to the remaining pure carbon. By heating the paint samples containing vine black, and knowing the relative weight loss of the pigment under pyrolysis (evaluated at about 4%), the pigment and the binder contents can be estimated. Of course in the case of the vine black, the evaluation is based only on heating under nitrogen and not oxygen (since heating under oxygen would result in the complete combustion of the paint sample). The third column shows the experimental results (weight lost) obtained in the presence of nitrogen in the TGA scans of the paint; in this case no correction was carried out.

The data clearly show that the paint reconstructions have almost 15% less binder than when the original paint samples were prepared eleven years ago. Although this can be partially attributed to the incomplete pyrolysis of the organic fraction taking place at 700°C, its extent was estimated to be under 5% in the paint reconstruction with lead white. The discrepancy between the original binder content of the paint with vine black and the current content must be maily attributed to the glycerol and free fatty acids evaporating from the paint film. Actually the phenomenon is more complex, as it is known that small weight changes in the early stages of drying occur due to the loss of small volatile compounds (ketones, aldehydes, etc.) and to a weight gain from the incorporation of oxygen [4], [28].

The different behaviours observed between the ZD and ZB series are the result of the effects of the two pigments. The lead in lead white in fact saponifies a substantial fraction of the binder, so when hydrolysis takes place, the fatty acids are transformed into the corresponding lead carboxylates. The thermal behaviour of lead carboxylates and free fatty acids is quite different, as can be inferred from the thermogravimetric curves of lead palmitate and palmitic acids, which show a higher weight loss at 350°C and 220°C, respectively (Figure 3): lead palmitate is much less volatile than palmitic acid. This indicates that the saponified oil medium is less subject to weight loss over time compared to the paint layer created with an inert pigment such as vine black.

#### *P/S ratios*

Some final remarks can be made regarding the P/S values obtained from the paint reconstructions with lead white and vine black (**Table 2**). The average P/S value of the ZD series is 1.4, with a CV of 7%. In the case of vine black, the average P/S value obtained is still 1.4, but the CV is higher: 12%. This is probably due to the fact that some of the free fatty acids from the paint film evaporated more in the vine black samples than in the lead white ones. In fact this evaporation is not perfectly homogeneous on the paint layer, since it is dependent on the paint thickness and the ratio pigment/binder of the samples collected from the paint film.

This result is extremely important to those dealing with diagnostic analyses of paint samples, for whom the P/S value represents a crucial parameter that has been used since over 50 years in the identification of the oil source in the paint binder [1] *and references therein*; [44] *and references therein*; [45] *and references therein* [46] *and references therein*. Considering that palmitic acid evaporates four times more quickly than stearic acid, the P/S value is expected to decrease with time [43] (this was not actually observed in this study, most likely as the paint reconstructions were only eleven years old). However, this study shows that a much bigger variability in the P/S ratio should be expected. It was in fact proven that the relative amounts of free fatty acids decreases considerably over time in a paint film in relation to the pigment present,. These results confirm the growing uncertainty among those working in the field relative to the reliability of the P/S parameter [12], [47], [48], [49].

There are thus other factors of fundamental importance in determining the final P/S value of a paint sample, some of which are the direct consequence of the fast evaporation of the free fatty acids from the paint film quantified in this study: the film thickness at the sampling point, the presence of any other organic materials that could hinder the evaporation of fatty acid as a consequence of the formation of non volatile complexes (for example protein-fatty acid complexes), the presence of pigments able to form complexes with the fatty acids [5], [12], [14], [15], [50], the presence of overlying layers [51], [52], the cleaning treatments [53], [54], and finally the thermo-hygrometric conditions of the storage places throughout the history of the paint [43]. On the basis of all this, the use of the P/S ratio as a parameter to determine the source of the oil should be carefully considered. If the pigments are able to saponify and thus stabilise all acids, probably the parameter can still be used. In the cases of pigments, such as vine black, which give no chemical stabilisation to the paint, the use of P/S can be misleading. Further research with other pigments and more prolonged ageing must be done to asses this aspect. Despite this, the influence of other parameters, such as the conservation and cleaning history of the painting, is extremely difficult to be quantified.

### The Role of the Different Pre-treatments Undergone by Linseed Oil

The thermogravimetric curves recorded under nitrogen and air flow of the ZD and ZB series with differently pretreated oils are reported in Figure 7, Figure 8, Figure 9 and Figure 10.

The thermogravimetric curves of the paint samples of the paint samples containing the same pigment are quite similar to each other, indicating that in all cases, hydrolysis, cross-linking and oxidation took place, as well as the formation of lead white compounds in the ZD series. Small differences are observed for the ZD series in the steps that take place above 400°C: there is a different number of peaks, showing a maximum at different temperatures and of a different broadness. This indicates that the oxidised and cross-linked fractions are present in all cases, however they are characterised by a different molecular weight distribution.

It is known that oil subjected to different pre-treatments will produce paints with specific rheologies [21], [22]. In a previous study we showed that most treatments were aimed, from a chemical point of view, at consuming the antioxidants naturally present, and part of the double bonds [28]. This then produced a partially cross-linked and oxidised oil, which was more polar than the untreated oil [25], [28]. These chemical modifications resulted in some oils that would dry faster than untreated oils, and would give better dispersions of polar pigments, thus influencing the handling properties of the paint [28]. Both the GC-MS and TG data strongly suggest that pre-treatments that produce oils with significant differences from the chemical and rheological point of view when they are still in the liquid form, do not significantly influence the ageing pathway of the paint layer. The heterogeneity observed by TG for the high molecular weight fraction of the ZD series seems in fact more related to non-homogeneous oxidation and cross linking, than anything else, due for example, to the proximity of the binder to the pigment particles and the paint surface.

### Conclusions

We have presented the first systematic investigation using GC-MS and TG of paint layers prepared with the same oil and processed according to various pre-treatments, using two different pigments: lead white, and vine black. This analytical approach characterised the oxidation and hydrolysis state of the binder and the presence of metal soaps in the lead white paint layers. A comparison of the data obtained from the paint layers prepared using the differently pre-treated oils, and the different pigments enabled us to draw several important conclusions:

*Double bond transposition*taking place during ageing is much more important than that due to the pre-heating of the oil.
As a consequence the *A/Sub and A/Seb ratios* are more influenced by the pigment than the pre-treatment undergone by the oil.
*Evaporation of the organic medium from the paint layers* takes place during ageing at a surprisingly fast rate, depleting the paint layers of glycerol and fatty acids. When fatty acids were saponified by the Pb^2+^ present in the lead white pigment, then their evaporation did not take place. Thus the presence in the paint layers of any material, pigment or other binder, which can form complexes or other sorts of chemical compounds with the fatty acids, hinders this phenomenon.
*The use of P/S ratio* to identify the plant origin of the oil [1] is not straightforward in an aged sample, because it is dependent on the sample age and composition, as an effect of the presence of other organic binders and pigments, conservation condition, cleaning history, etc.
The *P/S ratio* of an oil cannot be used to establish the sort of pre-treatment undergone by the oil.
Finally the data suggest that the pre-treatments that produce oils with significant differences from the chemical and rheological point of view when they are still in the liquid form, do not seem to significantly influence the ageing pathway of the paint layer. As a result *the composition of an aged oil paint* seems much more related to the pigments present and the conservation conditions than to the original pre-treatment of the oil. As a result the ratios between different amounts of fatty and dicarboxlic acids do not seem to be the analytical approach to be pursued to establish the pre-treatment undergone by the oil.
